# Maternal Health Situation in India: A Case Study

**DOI:** 10.3329/jhpn.v27i2.3363

**Published:** 2009-04

**Authors:** Kranti S. Vora, Dileep V. Mavalankar, K.V. Ramani, Mudita Upadhyaya, Bharati Sharma, Sharad Iyengar, Vikram Gupta, Kirti Iyengar

**Affiliations:** ^1^ Centre for Management of Health Services, Vastrapur, Ahmedabad, India; ^2^ Indian Institute of Management, Ahmedabad, India; ^3^ ARTH-3, Udaipur, India; ^4^ Population Foundation of India, Jaipur, India

**Keywords:** Delivery, Health indicators, Healthcare, Maternal health, Maternal health services, Maternal mortality, India

## Abstract

Since the beginning of the Safe Motherhood Initiative, India has accounted for at least a quarter of maternal deaths reported globally. India's goal is to lower maternal mortality to less than 100 per 100,000 livebirths but that is still far away despite its programmatic efforts and rapid economic progress over the past two decades. Geographical vastness and sociocultural diversity mean that maternal mortality varies across the states, and uniform implementation of health-sector reforms is not possible. The case study analyzes the trends in maternal mortality nationally, the maternal healthcare-delivery system at different levels, and the implementation of national maternal health programmes, including recent innovative strategies. It identifies the causes for limited success in improving maternal health and suggests measures to rectify them. It recommends better reporting of maternal deaths and implementation of evidence-based, focused strategies along with effective monitoring for rapid progress. It also stresses the need for regulation of the private sector and encourages further public-private partnerships and policies, along with a strong political will and improved management capacity for improving maternal health.

## INTRODUCTION

The World Health Organization (WHO) estimates that, of 536,000 maternal deaths occurring globally each year, 136,000 take place in India. Estimates of the global burden of disease for 1990 also showed that India contributed 25% to disability-adjusted life-years lost due to maternal conditions alone (1). Unfortunately, there is little evidence that maternity has become significantly safer in India over the last 20 years despite the safe motherhood policies and programmatic initiatives at the national level.

India, with a population of over a billion and decadal growth of 21% (Table 1), estimated its maternal mortality ratio (MMR) at 301 (maternal deaths per 100,000 livebirths) in 2003. (2) The MMRs vary across the states, with the large North Indian states contributing a disproportionately-large proportion of deaths. Uttar Pradesh and Rajasthan, for example, have high rates of fertility and maternal mortality while Kerala and Tamil Nadu have rates comparable with middle-income countries. Geographical vastness and sociocultural diversity across India contribute to this variation. The status of women is generally low in India, except in the southern and eastern states. Female literacy is only 54%, and women lack the empowerment to take decisions, including decision to use reproductive health services. As health services are governed at the state level, much also depends on state leadership and management skills.

**Table 1.** Demographic and health indicators of India and her states (2)

Indicator	India	Tamil Nadu	Gujarat	Rajasthan	Andhra Pradesh
Population (million) (Census 2001)	1,028	62	51	57	76
Decadal growth rate (1991-2001)	21	12	23	28	15
Population density per sq km (2001)	324	478	258	165	275
Birth rate (2005)	24	16	24	29	19
Death rate (2005)	7.5	7.4	6.9	7.0	7.3
Total fertility rate	3.2	1.7	2.9	3.7	2.0
Mean age (years) of effective marriage (2005)	20	22	20	20	19
Literacy rate: total (2001)	65.3	73.4	69.1	60.4	60.4
Male	75.3	82.4	79.9	75.7	70.3
Female	54.1	64.4	57.8	43.8	53.7
Sex ratio (no. of females per 1,000 males)	933	987	920	921	978
Life expectancy at birth—females (2005)	66	69	69	67	68
Infant mortality rate (2006)	58	37	54	68	57
Child mortality rate (2005)	17	9	16	20	15
Maternal mortality ratio as per SRS (2003)	301	134	172	445	195

SRS=Sample Registration System

The objectives of this case study were to describe the present situation of maternal health in India and its national safe motherhood programmes and analyze their impact. Suggestions are made to improve maternal health in the country.

## MATERIALS AND METHODS

Various methods were used for collecting relevant information, including a review of literature (i.e. published and unpublished reports of government and non-government agencies), secondary analysis of data from the management information system of national programmes and from states, interviews with stakeholders, and a study of key institutional processes, roles and authorities of key actors, organizational structures and functions, and administrative support. Data were also drawn from the National Family Health Surveys (NFHSs) and District Level Household Survey (DLHS). Information regarding health infrastructure and human resources was collected from the DLHSs, facility surveys, and national government documents/website.

Safe motherhood programme strategies and implementation, including past efforts and new initiatives, were analyzed to understand their effects on the performance indicators of maternal health. Reliable data on maternal mortality and morbidity in India, however, were not available, and the present estimates vary considerably. There were also gaps in information on the process and input indicators, such as number of functional First Referral Units (FRUs) for emergency obstetric care (EmOC) and availability of specialists.

## RESULTS

### Maternal mortality ratio and process indicators

#### MMR in India

The Government's Health Survey and Development Committee report of 1946, known as the Bhore Committee report, is one of the earliest references to maternal mortality in India. After reviewing the available evidence, the Committee concluded that the MMR in the country was around 2,000 deaths per 100,000 livebirths (3). The Mudaliar Committee estimated that the MMR had decreased to 1,000 in 1959 (4). A principal cause for the decline was thought to be the decrease in the incidence of malaria because pregnant women with malaria suffered higher fatalities (5). During 1984-1985, the first community-based study on maternal mortality in Ananthapur district of Andhra Pradesh gave an estimate of 798 for the district (6).

Results of more recent nationwide studies suggest that levels of maternal mortality have decreased (Table 2): a nationwide sample study in 1992 undertaken by the Indian Institute of Population Studies gave an MMR of 437 (7,8); estimates from the National Sample Surveys (NSSs) and the Sample Registration System (SRS) showed that maternal mortality declined from 1,300 deaths per 100,000 livebirths in 1957 to 301 in 2003 (4,9). Yet, the SRS and the vital registration system are considered to give underestimates, and an international experts group estimated the MMR to be 1.5 times the 2003 SRS estimate at about 450 (10). Regional estimates of maternal mortality based on small sample sizes, or estimates from the NFHS data, indicate that maternal mortality is much higher than that projected from the vital registration system. A range of estimates for the MMR for states is given in Table 3 (11-13).

**Table 2.** Estimates of maternal mortality ratio from different sources over the last 50 years (7)

Source of data	Reference year	Maternal mortality ratio
NSS, 14^th^ Round	1957	1,287
NSS, 16^th^ Round	1960	1,355
NSS, 19^th^ Round	1963-1964	1,174
SRS	1972-1976	892
SRS	1977-1981	844
SRS	1982-1986	568
PN Mari Bhat's estimate	1982-1986	580
World Health Report, 1999	1990	570
NFHS 1	1992-1993	437
1997-1998 retrospective MMR surveys	1997-1998	398
SRS	1997	407
SRS	1998	408
NFHS 2	1998-1999	540
SRS prospective household reports	1999-2001	327
World Health Report, 2005	2000	540
SRS special survey of deaths using RHIME	2001-2003	301

MMR=Maternal mortality ratio; NFHS=National Family Health Survey; NSS=National Sample Survey; RHIME=Routine, representative, resampled household interview of mortality with medical evaluation, a method used in SRS; SRS=Sample Registration System

**Table 3.** Regional variation of estimated MMR per 100,000 livebirths (11-13)

State	Source and year					
	Bhat∗	Bhat∗	IIHFW†	SRS	SRS	SRS
	1982-1986	1994	1998-1999	1998	2001	2003
Punjab	346	289	351	244	144	138
Haryana			468	161	190	169
Uttar Pradesh	879	612	737	867	772	700
Bihar			714	651	549	486
Rajasthan	614	588	526	647	655	561
Madhya Pradesh			601	554	534	474
Orissa			552	297	367	295
Assam	709	636	762	587	403	474
West Bengal			451	251	175	148
Maharashtra	414	471	365	172	138	117
Gujarat			393	52	199	166
Andhra Pradesh	379	383	341	151	176	148
Karnataka			364	225	229	189
Tamil Nadu			284	89	115	88
Kerala			262	92	93	66
India	580	544	466	348	312	274

∗ Regional estimates covering more than one state—based on rural households;

^†^ Estimates of MMR from a regression model based on the NFHS 2 data; IIHFW=Indian Institute of Health and Family Welfare, Hyderabad; MMR=Maternal mortality ratio; NFHS=National Family Health Survey; SRS=Sample Registration System

#### Causes of maternal mortality

**Fig. 1.**
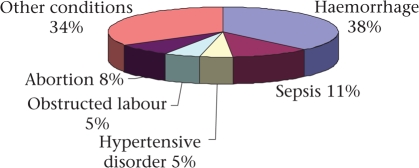
Causes of maternal deaths in India, 2003(14)

Haemorrhage is considered to be the major maternal killer in India: 38% of maternal deaths were caused by haemorrhage, mostly postpartum haemorrhage, according to a recent SRS analysis (Fig. 1) (14). Among ‘other conditions', anaemia was the main medical condition leading to maternal death. Anaemia, especially iron-deficiency anaemia, is highly prevalent among the Indian population: nearly 60% of pregnant women were anaemic, according to the 2006 NFHS (Table 4). Deaths due to sepsis and obstructed labour may be attributed to the high proportion of deliveries at home. Despite a liberal law on abortion in India, abortion-related complications cause an estimated 8% of all maternal deaths.

**Table 4.** Maternal health indicators (%) in India (15)

Indicator	NFHS 1 (1992-1993)	NFHS 2 (1998-1999)	NFHS 3 (2005-2006)
Pregnant women with anaemia	NA	50	58
Three antenatal check-ups	44	44	51
Institutional deliveries	26	34	39
Deliveries conducted by health personnel	33	42	48
Mothers received postnatal care within 2 months of delivery	NA	16	42

NA=Not available; NFHS=National Family Health Survey

#### Use of maternal health services

From the time of the first NFHS published in 1992 to the third and most recent NFHS (2006), the maternal healthcare indicators have slowly improved (Table 4). Institutional deliveries have risen from 26% to 39%, and nearly half of the women now have their births attended by health personnel. These figures represent a 1% yearly improvement in both the indicators over the 14 years separating the surveys. Postnatal care remains the most neglected area with only 42% of women receiving such care within two months of delivery, and a negligible number of women are visited in the vulnerable first week after delivery. These data underscore the overall slow progress despite the national safe motherhood programmes, such as Child Survival and Safe Motherhood (CSSM, 1992-1996), Reproductive and Child Health (RCH) phase 1 and 2 (RCH 1, 1997-2004, RCH 2, 2005-2010), and others discussed later.

#### Inequity in maternal health

**Fig. 2.**
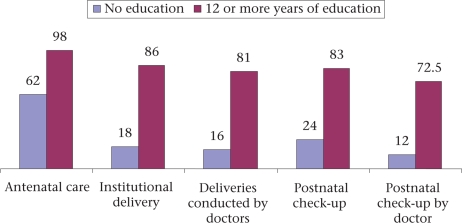
Access to maternal healthcare according to maternal education (NFHS 3, 2005-2006) (16)

**Fig. 3.**
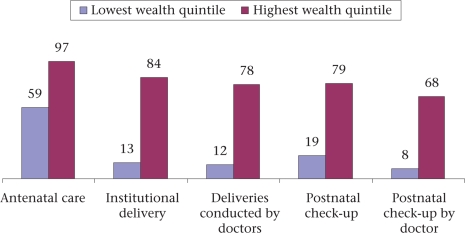
Access to maternal healthcare according to maternal wealth status (NFHS 3) (16)

The educational and economic status of women influences the use of maternal care. Figure 2 and 3 illustrate that illiterate mothers and mothers from the lowest wealth quintile used basic maternal healthcare much less than their literate or wealthier counterparts and were far less likely to see a doctor. Only 18% of 39,677 illiterate mothers had institutional deliveries compared to 86% of 39,677 mothers with 12 or more years of education; similar differences were observed in the use of skilled care at delivery and use of postnatal care.

NFHS=National Family Health Survey

NFHS=National Family Health Survey

Women of low economic status had availed 13% of institutional deliveries compared to 84% by women of the high wealth quintile. Only 19% of mothers of the lowest wealth quintile received postnatal care compared to 79% of mothers of the highest wealth quintile. These statistics reflect the inability of the public-health system to reach out to the poor and illiterate.

Despite the emphasis on antenatal care by the Government, about half of the pregnant mothers still do not complete three antenatal visits, and a quarter do not receive tetanus prophylaxis. The prevalence of anaemia increased between 1998-1999 and 2005-2006 to 58%, although the coverage of antenatal check-ups increased. During antenatal visits, iron-folic acid (IFA) tablets for anaemia are to be provided, and Table 5 shows that the percentage of women receiving any IFA tablets did increase: 65% of pregnant women stated that they received IFA tablets. The fact that the blood tests of more women showed anaemia, and more women received IFA tablets reflects the poor quality of antenatal care, poor nutritional status of women, and/or their poor compliance with taking IFA tablets (Table 4).

**Table 5.** Performance indicators (%) for maternal health services in India (17,18)

Indicator	India		
	NFHS 1 (1993)	NFHS 2 (1999)	NFHS 3 (2006)
Coverage of antenatal services			
Tetanus toxoid injection (2 or more)	54	67	76
Completed 3 antenatal care visits	44	44	51
Received IFA tablets	50	58	65
Place of delivery			
Institutional deliveries	26	34	40
Domiciliary deliveries	74	66	60
Institutional deliveries			
Public	15	16	18
NGO/trust	NA	0.7	0.4
Private	11	17	20
Type of deliveries			
Vaginal deliveries	97	93	91
Caesarean sections	3	7	9
Assistance during delivery			
Doctor	22	30	35
ANM/nurse/midwife/LHV	13	11	11
Other health professionals	NA	1	1
Dai (TBA)	35	35	37
Other	30	23	16

ANM=Auxiliary Nurse Midwife; IFA=Iron-folic acid; LHV=Lady Health Visitor; NA=Not available; NHFS=National Family Health Survey; NGO=Non-governmental organization; TBA=Traditional birth attendant

Institutional deliveries rose from 34% in 1999 to nearly 40% in 2006. However, the percentage of deliveries taking place in public institutions has not shown remarkable improvement; most increase is in the private sector where about half of institutional deliveries now take place. The caesarean-section rate has also increased and is now 9% nationwide. Most increase was seen in urban India, and in private facilities; access to comprehensive obstetric care continues to be a problem for rural women. ‘Increased assistance by a health professional' has meant that more women rely on doctors for their deliveries: about 35% of women in 2005 sought assistance from doctors for birth—up from 30% in 1999. The proportion of deliveries by traditional birth attendants (TBAs) remained steady at a little more than one-third over the past decade and a half, with a slight decline in the percentage conducted by an Auxiliary Nurse Midwife/Lady Health Visitor (ANM/LHV). Altogether, nearly 50% of women now seek professional care at delivery (Table 5).

### Maternal healthcare-delivery system

#### Facility-level maternal healthcare

Although statistics from rural areas show less use of services than urban areas, the Indian Government has focused on rural healthcare since independence. Post-independence India developed a three-tier healthcare-delivery system to reach out to remote areas to provide primary care at the village level, secondary care at the subdistrict and district levels, and tertiary care at the regional level. Medical colleges were developed as apex institutes with specialities. Over the 50-year period since independence, India has expanded the public-health infrastructure to include 144,988 Subcentres (SCs), 22,669 Primary Health Centres (PHCs), and 3,910 Community Health Centres (CHCs) (Table 6).

**Table 6.** Details of public-health facilities, 2006 (19-21)

Healthcare institution	Population norms	Level	No. in India (2007)	No. in India (2006)	Highest medical services provider
Medical college hospital	5-8 million	Apex	242	242	Super specialists
District hospital	2-3 million	III	370	370	Specialists, including obstetrician
First Referral Unit	3,00,000-5,00,000	II	1,762	1,926	Obstetrician
Community Health Centre	1,00,000-3,00,000	II	4,045	3,910	Medical officer/specialists
Primary Health Centre	30,000	I	22,370	22,669	Medical officer, staff nurse
Subcentre	5,000	I	145,272	144,988	Auxiliary Nurse Midwife

Even with this emphasis to create infrastructure, the Indian public-health system is fraught with basic problems. For example, there is no system of accrediting health facilities or evaluating functionality of health facilities at the state or national level. Table 7 shows that over half of the SCs and 30% of the PHCs do not have their own buildings. About a quarter of the FRUs do not have telephones, and 40% do not have a vehicle. Over 70% of the FRUs and CHCs do not have linkages with a district blood-bank. More than half of the CHCs, FRUs, and district hospitals do not have residential quarters for staff. None of the national facility surveys mentions the maternity ward; so, it is not clear how many exist.

**Table 7.** Infrastructure and human resources available (%) in India for maternal healthcare, 2006 (22,23)

Infrastructure	Subcentre	PHC	CHC	FRU	DH
Own building	45.2	69	84	94.7	97
Electricity	43.1	66.4	91.8	94.3	96.7
Operating theatre	NA	NA	87.6	93.7	99.5
Labour room∗	NA	48.4	31.0	33.3	44.4
Telephone	NA	NA	62.2	74.8	96.7
Vehicle on road	NA	NA	57.4	56.8	89.9
Linkage with district blood-bank	NA	NA	15.8	27.2	67.5
Quarters for RMO	NA	NA	44.0	42.2	47.1
Obstetrician	NA	NA	51	71	90.0
Anaesthesiologist	NA	NA	37	69	83.0
Paediatrician	NA	NA	54	73	90.0
Staff nurse	NA	NA	83	88	90.0

∗For CHC, FRU, and DH, information is available for separate aseptic labour room; CHC=Community Health Centre; DH=Department of Health; FRU=First Referral Unit; NA=Not available; PHC=Primary Health Centre; RMO=Registered Medical Officer

Table 7 also shows the dearth of a skilled staff, particularly specialist, for providing EmOC. About 50% of the CHCs and 30% of the FRUs do not have anaesthetists, and the same percentages do not have an obstetrician; it is not known how the needed team of obstetrician and anaesthetist is available for emergency care.

#### Community-level maternal healthcare

After independence in 1947, rural health services were established over time with primary health units (PHUs) serving a population of 30,000. Trained nurse-midwives were posted in hospitals or PHUs to provide maternal health services. SCs were established below the PHUs to provide basic medical care and delivery care at the field level. Temporary workers—local women with a primary education—were recruited and trained for a short period to man the SCs. They were called ANMs. According to the World Health Organization (WHO), auxiliary workers are technical workers in a particular field with less than full qualifications. The Shetty Committee suggested training auxiliary nurses and midwives for a short period to work under supervision for specific duties (24). Over time, various committees, such as Mudaliar, suggested the continuation of auxiliary cadre to provide basic healthcare at the field level (4). The ANMs gradually became permanent staff in the public-health system.

Initially (1960), the ANM was envisaged as a community-based midwife to provide various public-health services with focus on maternal and child health. The ‘Multipurpose Health Worker Scheme', introduced in 1975 following the Kartar Singh Committee's recommendation (25), converted the ANM to a multipurpose health worker looking after primary healthcare, including disease control. In 1977, a second policy change integrated maternal and child health (MCH) into family planning, renaming it ‘Family Welfare'. These two policy changes resulted in a drastic decline in the quality of the ANMs' midwifery training and practice in the country. Under pressure from the Government, the Indian Nursing Council (INC) revised the ANM course and reduced its duration from 24 months to 18 months in 1977. Along with this dilution and weakening of the ANM's skills, her technical supervision also underwent a decline (26). By the end of the 1970s, community perception of ANMs also changed from MCH care providers to family-planning/immunization workers.

### Management of maternal health services

At the national level, there are two major divisions within the Ministry of Health and Family Welfare: the Department of Family Welfare (DFW) and the Department of Health (DH). MCH, reproductive health, rural health, primary healthcare, and family planning come under the DFW while medical colleges, national institutes, and disease-control programmes come under the DH.

The Maternal Health Division within the DFW looks after all technical and administrative aspects of maternal health activities throughout India (Box).

Box. **Functions of Maternal Health Division, Department of Family Welfare, India**

- rovision of technical advice to the Minister and the Secretary of Health and Family Welfare who are non-technical officials
- Designing new evidence-based maternal health programmes
- Setting technical standards and developing guidelines
- Reviewing research and developing new evidence-based strategies
- Reviewing training content and tailoring it to emerging needs
- Monitoring programme, implementation, and performance, including quality and evaluation of outcomes
- Providing information to address questions in the parliament
- Providing technical information on policy, legal and other issues
- Commissioning special studies and reviewing data
- Dealing with professional organizations, non-governmental organizations, consumer groups, etc.
- Interacting with donors, international agencies, and development partners
- Planning and implementing national information, education, and communication
- Preparing budgets and funding programmes

Given its multiple functions, it is clear that the Maternal Health Division needs a high level of technical and managerial capacity. Yet, the Division is composed of only four officers—one Deputy Director General (DDG) of maternal health, and three Assistant Commissioners of maternal health (one of these Assistant Commissioner posts has been vacant for more than 10 years). This structure has not changed over time: the annual reports of the Ministry for 1998-1999 showed the same structure. The present structure of the Maternal Health Division with only three officers is highly inadequate, not just in terms of numbers but also in terns of training and skills. They have no decision-making powers, and it is not compulsory for them to have public-health training or specific qualifications in maternal health. Any officer from the Central Government Health Services (A healthcare system for curative services established for Central Government employees) can be assigned to the Maternal Health Division. As all technical officers come from the Central Government Health Services that are mainly in Delhi and other small union territories, the officers typically do not have much field experience of implementing programmes at the state level. They can be transferred in a short time as they do not have a fixed tenure in the Maternal Health Division; this affects their performance as there is a learning period in every position. The officers of the Maternal Health Division reported that they spend about 40-50% of their time on non-technical issues; more time is used in administrative work because the lower-level administrative support is also weak.

Managerial capacities at the state level for maternal health are also a major problem. No state has a dedicated officer for maternal health, and most states have only 2-3 officers looking after all activities of MCH/RCH (27).

### Safe motherhood programmes in India

#### Child Survival and Safe Motherhood and Reproductive and Child Health Projects

During the mid-1970s, immunization received high priority, and the Expanded Programme on Immunization (EPI) for children aged less than five years was initiated. Vaccination tasks were assigned to the ANM. By 1992, the immunization programme in India evolved into the national CSSM programme, developed by the Government of India and supported by the World Bank and United Nations Children's Fund (UNICEF). It was designed to provide both child survival (e.g. immunization, diarrhoea, and acute respiratory infection control) and safe motherhood services (e.g. setting up FRUs, tetanus immunization, prevention of anaemia, antenatal care, delivery by trained personnel, including trained traditional birth attendants (TBAs), promoting institutional deliveries, and birth-spacing) through the PHC system in India. Of its eight goals, one was for maternal health, viz. reduction of maternal mortality from 4 to 2 per 1,000 livebirths. Although the package specified care at birth as a service, the workplan of ANMs at the SC did not specify conducting deliveries in the list of critical activities. Similarly, this was missing from the module for planning MCH services at the PHC and SC levels and the sample workplan of ANMs given in the workers' manual (28). Although the ANM's duty as a community midwife finds mention in the policy documents, it was not implemented in the field. The programme also created a conflict through its fixed-day scheduling of work for ANMs by giving more priority to routine preventive services, such as immunization and antenatal care, compared to emergency services, such as delivery care.

Following the International Conference on Population and Development in 1994, the Government started the process of re-orienting the family-planning and MCH programmes into a new one—the RCH 1. The RCH programme added further interventions to those of the CSSM, including treatment of reproductive tract infections (RTIs)/sexually transmitted diseases (STDs), establishment of blood-storage units, referral transport, and access to safe abortion. To provide skilled care at birth, the RCH programme incorporated additional nursing staff for the PHCs for round-the-clock maternal health services and staff incentives for night-time institutional deliveries. All these new efforts were added without increasing human resources in management at the central or lower levels. Key features of the maternal health component and issues in implementation of the CSSM and RCH programmes are presented in Table 8.

**Table 8.** Key elements of maternal health component of CSSM and RCH 1 and issues in implementation (29,30)

Key elements	CSSM	RCH 1
	Duration: 1992-1996	Duration: 1997-2004
Strategies	Upgrade existing CHCs to FRUs for providing EmOC Convert village-level immunization to mother and child-protection sessions Train TBAs and upgradation of skills of existing staff Provide ANMs with subcentre medicine-kit Educate people about the programme Provide equipment/supplies for safe motherhood and neonatal care at the CHC level	Make FRUs functional by providing contractual staff, building renovation Increase availability of specialists Ensure availability of blood at FRUs Provide funds given to local governing bodies to provide emergency transport facilities Improve quality of services Provide additional honoraria to PHC and CHC staff for attending deliveries after office hours Engage additional staff nurse for selected PHCs for 24 hours x 7-day delivery services Provide mode of transportation for ANMs Provide fixed drug and equipment-kit at each level as given in CSSM
Service package	Immunization of pregnant women Prevention and treatment of anaemia Antenatal care and early identification of maternal complications Delivery by trained personnel (including trained traditional birth attendants) Promoting institutional delivery Management of obstetric emergencies Birth-spacing	Essential obstetric care EmOC 24-hour deliveries at PHC and CHC Referral transport Blood storage at FRUs Access to medical termination of pregnancy
Issues in implementation		
Training	Short-term (6 days) training of MOs with little focus on maternal health Long-term training for EmOC skill-building of general doctors was not implemented Supplies and infrastructure improvement did not link with training	Training load could not be completed A few medical officers trained in short course for anaesthesia and resuscitation for EmOC which was not enough for skill-building Training modules developed along with National Institute of Health and Family Welfare but practical training too short (2 weeks) for skills development
Supplies	Delayed supply of high-quality useful equipment Low use Maintenance system not developed	No flexibility for local purchase of required supplies Lack of supplies and equipment No maintenance contracts
Staffing	Dearth of key staff/specialists at FRUs—making them dysfunctional No additional staff recruited General doctors and ANM/nurses lacked skills in EmOC	Private anaesthetist and obstetricians not available in remote areas on contract The role of ANMs and staff nurse hired on contract was not clear and insecurity of job
IEC and community participation	Limited scope and coverage of programme Limited to essential obstetric care	Communication and awareness about the programme preceded improvement in service-delivery which led to dissatisfaction with the system
Service-delivery	Safe motherhood component was partially implemented or remained weak Access to blood at FRUs was difficult because of high standards Proposed maternal mortality review committees not established	Inadequate linkages between components, such as family planning, maternal health, child health, and RTI/STD Haphazard implementation, e.g. some FRUs got additional staff while others got renovated; some villages received the transport-money; and others did not Transport-money remained unused Contractual staff did not provide round-the-clock services No efforts towards improving quality of services Process for licensing blood-storage unit at FRUs too long
Supervision, monitoring, and evaluation	Data not available for all components of the service package Safe motherhood focused only on TT coverage and IFA distribution Functionality of FRUs not monitored, no service statistics for FRUs collected Modified the existing management information system, but more focused on immunization and family planning	Independent district-level household surveys commissioned to assess RCH services A few components were closely monitored; anecdotal evidence indicates large-scale inflation of service statistics by field functionaries Functionality of FRUs continued to be unmonitored Systematic comprehensive evaluation not done

ANM=Auxilliary Nurse Midwife; CHC=Community Health Centre; CSSM=Child Survival and Safe Motherhood; EmOC=Emergency obstetric care; FRUs=First Referral Units; IEC=Information, education, and communication; IFA=Iron-folic acid; MOs=Medical officers; PHC=Primary Health Centre; RCH=Reproductive and Child Health; RTI=Reproductive tract infection; STD=Sexually transmitted disease; TBAs=Traditional birth attendants; TT=Tetanus toxoid

### RCH 2 and National Rural Health Mission

In 2005, with the assistance of World Bank and other donors, the RCH 2 programme was started as a follow-on to the RCH 1 programme and placed under a new government initiative—the National Rural Health Mission (NRHM). The NRHM, a seven-year (2005-2012) initiative to increase public-health spending, has provided substantial additional funding and given high priority to revamping rural health systems. It intends to bring all health and family welfare and allied sector programmes under one umbrella to improve the health of the rural people and provide a further thrust to reduce child and maternal mortality and fertility. The RCH 2 programme has led to decentralization, flexibility in programming, and increased financial allocations to field-level workers. One priority area of the NRHM is to have a female health volunteer (FHV) in each village—called Accredited Social Health Activist (ASHA)—in some sense reviving the village health worker scheme of the 1970s. Other efforts are being made to provide quality reproductive health services, including institutional delivery, safe abortions, treatment of RTIs, and family-planning services, to meet unmet needs while ensuring full reproductive choice to women.

Under the NRHM, the main strategy of the Government for reduction in maternal mortality focuses on institutional deliveries and provision of EmOC. The Government has recently changed policy to allow staff nurses and ANMs to initiate treatment of pregnancy-related complications, including intravenous fluids and injectable Oxytocics, antibiotics, and magnesium sulphate—all earlier restricted to administration by doctors. The Government has also started the retraining of ANMs to improve their skills as skilled birth attendants (SBAs). The Central Government has encouraged a 16-week training of MBBS doctors in anaesthesia and comprehensive EmOC. Information, education, and communication (IEC) efforts are also being intensified to focus on maternal health interventions. The CHCs are being upgraded to FRUs for providing referral services to mothers and children, care for obstetric emergencies and complications, and provision of safe abortion services (31).

### Blood-banking services: vital but neglected

Blood-transfusion services occupy a vital space in any national health service-delivery system, but not so in India. Most government blood-banks in India operate in hospitals with minimal infrastructure and inadequate/irregular supply of blood. A Supreme Court Judgement in 1996 resulted in improvements in blood safety in India but it also made blood even scarcer in rural areas as more than necessary infrastructural requirements were mandated for licensing blood-banks. In response to the need for more safe blood in rural areas, the Central Government, in a policy change in 2001, stated that small blood-storage facilities were to be developed at the FRU level. The aim of the ‘blood-storage unit' is to procure tested and safe blood from a city blood-bank, store it, and provide it after cross-matching to patients who need it in the rural FRUs. For the blood-storage unit to function efficiently, there must be surplus blood available at city blood-banks and a mechanism to compensate the cost of collection and testing of blood. This ‘give and take' of blood also needs trust and understanding between the blood-bank and the blood-storage unit. Only a few blood-storage centres are now operational, and most comprehensive EmOC centres continue to have no connections with blood-banks (32). The recent initiative by the National AIDS Control Organization will help develop blood-transfusion services and reorganize them to make safe blood available to those in need but this process is very slow and highly under-funded.

There are no monitoring or data on indications of blood transfusions and complications of blood transfusions. It is difficult to determine the availability and use of blood in obstetric emergencies. Blood is free but patients have to pay for processing charges (about US$ 10-15 per unit). This is a major barrier to accessing blood by poor women who need it, especially in emergency. Further details are delineated in the paper on blood-banking services in this issue of the Journal (33).

### Referral transport and communication

Emergency transport and communication is an important part of all emergency health services, specifically for reduction of maternal mortality. Unfortunately, the Government of India never planned any systematic intervention to improve the use of the communication and emergency transportation system for healthcare in the country. States have been buying ambulances on an ad-hoc basis as and when budgets are available; however, their maintenance and management are grossly neglected. Telephones were provided in the larger hospitals but were not available in the PHCs until recently. Unfortunately, even when telephones are available, no protocols were developed for how to use them for improving emergency referral. Advanced communication technology in recent years has, to an extent, alleviated the problem since most healthcare providers now carry personal mobile telephones. Recently, some state governments are also providing mobile phones with free calling in the closed user group (CUG) of staff of the Health Department of the Government.

### Financial resources

The health system of India has been chronically under-funded for the last 40 years. The Government spends only about 0.9% of gross domestic product on health services, including expenditure by the Central and State Governments, one of the lowest in the world. The CSSM and RCH programmes contributed an additional fund of US$ 600 million, about US$ 300 million of which went to maternal health, spread over 12 years. However, during these 12 years, there were about 300 million new births in India, giving an average of additional US$ 1 per birth, which is insufficient to really change maternal care provided to pregnant women. Given the huge size of India, it cannot hope to improve maternal health based only on donors' support. The Indian states bear the primary responsibility for health as per the constitution and must increase their funding substantially.

Compounding this low level of funding, the financial systems of the Indian Government are highly bureaucratic, slow, and procedure-oriented, resulting in non-availability of funds at peripheral locations where needed even when money is centrally available, remaining unused funds, and hence funds are lapsed after the financial year is over. The financial and audit rules require much procedure and paper work for using money budgeted. Under the NRHM, the Government is trying to streamline this process. As many states of India are in a severe financial crisis due to reluctance to collect taxes and profligacy of expenditure in the Government, this affects funds for maternal health.

### Innovations for maternal health services

Anecdotal evidence from states suggests that there has been a major increase in institutional deliveries because of the financial incentives (*Janani Suraksha Yojana*) (34) and ASHA programmes. Some individual states have also implemented innovative schemes for maternal health in the past few years that have shown good results (7,35,36).

#### Improving access, use, and quality of EmOC

- The Government of India, in collaboration with the Averting Maternal Deaths and Disability (AMDD) project, White Ribbon Alliance India (WRAI), Centre for Development and Population Activities (CEDPA), Johns Hopkins Program for International Education in Gynecology and Obstetrics (JHPEIGO) and other partners, has developed several guidelines to help improve EmOC. These include (a) a four-month competency-based curriculum and training system for training MBBS doctors to provide anaesthesia and comprehensive emergency obstetric services in rural areas, (b) SBA guidelines for medical officers, staff nurses, and ANMs, (c) guidelines for blood-storage unit, and (d) guidelines to operationalize the FRUs.
- The *Janani Suraksha Yojana* (Women's Protection Scheme), implemented in 2006 under the NRHM, promotes institutional deliveries by upgrading the National Maternity Benefit Scheme. It is exclusively funded by the Central Government and aims at improving delivery and post-delivery care for poor women, especially in rural areas. It offers a cash incentive for nutrition and transport for institutional deliveries to women undergoing delivery in government institutions and selected private institutions. This has resulted in a steep increase in institutional deliveries in several states according to anecdotal evidence and service statistics. States are given flexibility of managing the scheme at the local level. The financial and management guidelines are clear and available online. The Central Government is closely monitoring this scheme, especially in low-performing states, such as Uttar Pradesh and Bihar (34).
- The ASHA scheme, launched in some states, has improved community-mobilization efforts significantly by linking the community through a local volunteer with the government field workers and with facilities for institutional delivery. It is the role of ASHA to identify the pregnant women to make sure that they receive adequate antenatal care, natal care, and postnatal care.
- The Indian public health standards (IPHS) have proposed for the first time some basic standards for each level of health facility—where maternal health services are being delivered. This includes minimum equipment for labour rooms, sterile conditions, etc.

### Improving human resources and logistics management

- The Government of India and the Federation of Obstetricians and Gynaecology Societies of India (FOGSI) have started a 16-week training for general doctors for comprehensive EmOC in selected states since 2005. This training has been expanded to the national level to help overcome the gap in health professionals available to manage delivery-related complications. State-level initiatives by Gujarat and some other states have trained general doctors on anaesthesia. This effort, albeit in the right direction, requires scaling up and efforts to ensure that these doctors actually help deliver of emergency services in rural areas.
- Under the RCH 1 and 2 programmes, additional staff nurses have been provided to the PHCs to help conduct deliveries.
- The Tamil Nadu Medical Services Corporation, a public body, and the *Rogi Kalyan Samitee* (RKS), a local trust involving both community and facility leaders, have improved management of logistics by ensuring available supplies of essential drugs and equipment and their maintenance and decentralizing financial authority to improve day-to-day management at primary healthcare facilities. Now, all states are to start RKS at the CHCs and district hospitals to generate small income to improve their infrastructure. This is part of the World Bank-initiated cost-recovery efforts.

In addition to these efforts, many NGOs work on safe motherhood in India, in collaboration with the Government, and have carried out successful pilot innovations to improve maternal health indicators.

## DISCUSSION

Given the multiple efforts of the Indian Government to improve maternal health, why have the maternal health indicators not improved significantly in the past decade? The CSSM and RCH 1 programmes were each planned and implemented with separate, but connected schemes to strengthen the delivery of health services to reduce infant and maternal mortality. Unfortunately, due to lack of the managerial capacity, clear overall programme objectives, and evidence-based strategies, the schemes were implemented as an unconnected patchwork of efforts and, hence, did not lead to the desired improvement in the performance and achievement of objectives of the health system. Even the project completion report of the World Bank for the RCH 1 programme rated it as unsatisfactory (37).

Under the CSSM and RCH 1 programmes, the key strategy of operationalization of FRUs was not possible because the Government was unable to depute obstetricians and anaesthetists to rural areas. Second, the Government did not seriously attempt to train general (MBBS) doctors to provide comprehensive obstetric care and anaesthesia under these two programmes. Thus, there were failure of governance and lack of planning and implementation of a well-known strategy of delegating life-saving functions to available human resources (medical officers and nurses). Third, under the CSSM programme, the supply of equipment was substantially delayed due to financial and procedural problems. When the equipment was provided, it remained unused due to lack of training, motivation, monitoring, and maintenance. All the above failures could be traced to lack of the management capacity in the Government at the national and state levels.

The RCH 1 programme introduced some innovative experimental solutions to long-standing problems. However, instead of piloting them on a small scale and perfecting these solutions before scaling up, they were applied across the vast country. This led to highly uneven results and substantial failure. For example, the non-availability of anaesthetists and obstetricians was to be addressed by hiring a private specialist on a fixed payment per case basis for emergency caesarean sections. The assumption was that private specialists would be available in adequate numbers in rural areas where needed and would be willing to work with the Government at the fixed rate of compensation being provided, and the government system would be able to manage such contracts. This scheme did not work well because all these assumptions were not realized in many parts of the country.

One scheme under the RCH programme was to pay additional honoraria for deliveries conducted after office hours (5 pm to 8 am). This scheme ran into problems as, in some districts, the number of deliveries at night suddenly shot up, indicating probable falsification of data to get additional honoraria by the staff. The provision of additional ANMs hired on contract in selected PHCs to provide delivery services seemed to work only in some areas. Whether posting one additional staff nurse in the PHC increased the number of deliveries conducted by the institution is not known as there are no data collected to monitor this scheme.

### Other reasons for limited success of programmes

#### Lack of reliable estimates of maternal mortality

Establishing a reliable vital registration system is a must to achieve low rates of maternal mortality; without it, the impact of safe motherhood programmes remains unknown. Sweden, Sri Lanka, and Malaysia established robust vital registration systems at early stages of their battle against maternal mortality. Information on the levels, causes, and patterns of maternal mortality in India is, at best, incomplete and unsatisfactory compared to infant mortality for which estimates are available from the Registrar General of India. There is a lack of sufficiently-robust systems for estimating maternal mortality routinely either in the SRS, vital registration system, community-based surveys, or hospital-based data. The negligence to the issue itself is perhaps, indicative of the position accorded to women in India.

#### Absence of independent advocates for maternal health in civil society

Maternal death has not been a subject of sociopolitical or legal debate in India. Professional medical organizations have tried to promote maternal health but have made little impact as collaboration between the professional bodies and the Government on maternal health issues has been weak. Women's NGOs that have actively blocked the introduction of injectable and implant contraceptives ostensibly in the interest of women have, however, neglected the tragedy of maternal mortality. Political parties and leaders have not played an active role in promoting maternal health (38). International agencies, such as World Bank, United States Agency for International Development, UNICEF, and United Nations Population Fund, have focused on family planning and child survival (UNICEF), neglecting maternal health. As maternal death and disability do not cause any obvious epidemic, even the mass media hardly pay any attention to this tragedy. Consumer groups, the judiciary, and members of legislative assemblies and of parliament also ignore the massive tragedy of maternal mortality. The National Human Rights Commission and the National Women's Commission have paid little attention to the high number of maternal deaths in India.

#### Human resources

While some efforts have been made to address the human resources issue in maternal health services by increasing their numbers (e.g. three PHC nurses now allow 24-hour x 7-day service in Tamil Nadu), adding more tasks to the workload of existing staff or task shifting (e.g. ANM), or contracting with the private sector (39), there remains a dearth of available skilled manpower and mismanagement of available human resources, especially in the rural public-health system.

According to field observations, small studies, and interactions with stakeholders, a major issue is the motivation of staff and their commitment to overall health objectives. For example, we have seen health centres with an obstetrician and equipment but little performance. In states where private practice is allowed to government doctors, there could be a financial incentive to under-perform in the government facility so that patients can be diverted to their private clinics. With almost no regulation of the private sector, weak monitoring of public-health institutions and questionable moral and ethical standards among health professionals, it is not difficult to imagine that financial self-interest of doctors in the form of private practice may lead to low standards of care and attention in the public system.

Another example of motivation concerns the ANM in her role as both family planning and MCH care provider. As monitoring at a higher level concentrates on family-planning indicators, these peripheral health workers focus on provision of family-planning services only. Even in the NRHM where the emphasis is on institutional deliveries, there is little pressure on peripheral workers to provide delivery care and no incentives for this.

That the compensation package and promotional opportunities typically do not depend on performance of staff are further factors that impact motivation of workers. An extreme example is the ANM or doctor, who lives in a remote village and provides 24-hour services, gets the same salary and benefits as an ANM or a doctor who lives in a city, and commutes irregularly to a rural health centre for a few hours a day where s/he is posted.

Absenteeism or not living at place of posting is rampant (40). The government care providers typically want to live and work in larger cities for the comfort of their families and for educational and other opportunities; or they may not stay at a rural posting making round-the-clock delivery and emergency services unavailable. Not staying at the place of posting affects delivery care and EmOC more severely than primary preventive interventions, such as immunization, family planning, etc. As posting in remote places may be a punishment for poor-performing staff, a staff member posted in a remote area may be of low calibre. Unclear policies for posting and transfer have allowed scope for personal and political influence to obtain preferred postings.

Another policy loophole has been around delegation of clinical functions at the field level. Until recently, nurses and ANMs were not allowed to provide basic EmOC, and medical officers were not allowed to provide comprehensive EmOC in the absence of specialists (41) **)**.

#### Lack of focus on institutional deliveries

Total institutional deliveries remain less than 40%, and as of 2004-2005, the public-health institutions together (PHCs, CHCs/rural hospitals, district hospitals, and teaching hospitals staffed by specialists, medical officers, and nurses) conducted only 16% of total number of deliveries (NHFS 2) (Table-5). The increase in institutional births between 1993 and 2006 was primarily in the unregulated private sector. With the maternity benefit scheme—*Janani Suraksha Yojana*, this is now changing, and many states are reporting rapid increase in institutional deliveries at such a level that they are unprepared. In some facilities of Rajasthan, for example, there are more than one woman per bed because of the increased demand for institutional births.

### Lessons from high-performing states and innovative programmes

Kerala and Tamil Nadu have consistently reported low maternal and child mortality. The key factors for success in Kerala have been high political commitment for social sectors, high level of awareness in the community (e.g. the majority of women have a higher education), primarily an urban population, and good infrastructure (roads) leading to high access to public-health services. The government and private health infrastructure (health centres and hospitals) are much better in terms of numbers, density, and, perhaps, accountability. This combined with high awareness in the community has led to high use and better health outcomes in Kerala.

In Tamil Nadu, the recent decline in maternal mortality has been largely due to a series of initiatives taken by the State Government described earlier. The key lesson from success in Tamil Nadu is a long-term focus on maternal mortality through pilot-testing of evidence-based interventions on a smaller scale and then upscaling successful ones, with focus on the systematic implementation of interventions suited to local conditions to provide consistent higher-quality services in rural areas. Making higher investments, such as posting three nurses to a PHC and giving Rs 6,000 as a cash incentive for institutional delivery to poor women (a scheme similar to *Janani Suraksha Yojana*), are measures that are far ahead of what other states are willing to do. In addition, monitoring maternal deaths, analyzing medical and social causes, and taking actions to improve the system are all largely possible because of consistent and highly-committed leadership provided by technical officers in the health department over the years.

The success of the Chiranjeevi Scheme in Gujarat is largely due to the widespread availability of the private obstetrician-gynaecologist in rural areas and their willingness to collaborate with the Government. The Government made a credible and practical scheme of contracting out delivery services to the private sector through detailed consultations, planning, and transparent financial arrangements. Trust between the public and the private sector was created through the personal contact of the Health Commissioner and his team with private care providers in rural areas. The success of the training of medical officers is largely due to the increase in the management capacity created by appointment of a dedicated qualified official to oversee and manage the programme. The success of Gujarat also is driven by the committed health commissioner with education in public health and strong leadership who has been in the same position for the last four years.

### Recommendations for improving the effectiveness of future safe motherhood interventions

To ensure that the safe motherhood agendum is not neglected in the future, the following points need to be considered for any safe motherhood initiatives for reduction of MMR in India:

### Addressing policy, programme priorities, and governance issues

#### Improved management capacity and human-resources development

For any programme to succeed, four critical inputs—(a) resources, (b) management structure and systems, (c) correct strategy, and (d) an efficient implementing organization—are needed. Resources are always a constraint in any developing country. India has a large implementing organization in the form of the public-health system in rural areas, although its quality and accountability are major issues. The lack of progress in reducing the MMR can be attributable to incorrect strategies selected and lack of focused efforts in India. Choosing a correct strategy is a top management function. If the top management capacity is inadequate, selection of the right strategy becomes difficult. The management capacity at the district, state and national levels should be enhanced by management skills training for existing officers and recruitment of public-health professionals.

For public-health and facility-based staff, there should be clear policies for posting and transfer of staff, delegation of authority, and accountability. The objective of the policy should be to provide high-quality 24-hour delivery and emergency obstetric services. Development of skills and empowerment of medical officers, nurses, and midwives for EmOC services even in the absence of an obstetrician should be quickly and effectively implemented within a functional referral system. An attempt should be made to decentralize clinical life-saving skills, including elements of basic EmOC, from doctors to nurse-midwives to increase access to maternal health services across widely-dispersed rural populations. Care by midwives in rural areas should be linked through formal referral transport to emergency obstetric services within hospitals. The performance of each service provider should be reviewed with a focus on maternal health services.

#### Evidence-based and focused strategy for reducing MMR

Past experience has shown that attempting too many interventions with limited managerial capacity does not lead to success. Future programmes should, therefore, focus on specific, evidence-based strategies, such as skilled birth attendance, referral, and EmOC. This, in itself, is a challenging task in the extensive and varied infrastructure of the Indian health system. The PHCs and CHCs should perform all basic EmOC functions while the FRUs and district hospitals should provide comprehensive EmOC on a 24-hour x 7-day basis.

#### Annual implementation plans and monitoring progress

The state governments need to prepare annual plans to operationalize the FRUs and increase skilled birth attendance. These plans should be closely monitored to measure progress. Supervision and monitoring should include assessing the functioning of the facilities and their output and quality, based on appropriate indicators, such as the process indicators of the United Nations for EmOC.

### Improvements in coordination

Efforts to improve maternal health should ensure that all critical inputs, such as staff, drugs, blood, and equipment, are coordinated, provided, and monitored at strategically-selected locations in a timely manner for achieving the objectives. There is a need for coordination not only in the different arms of the Ministry of Health and Family Welfare but also between different government and non-government agencies.

### Improved public-private partnerships

Public-private partnership is on the agenda of the Indian Government to improve maternal healthcare but a uniform process has not been identified. There are no guidelines for public-private partnership for maternal health services. The majority of institutional deliveries take place in the private sector, and there is a need to regularize this sector to ensure the quality of care without causing further hardship to the poor. Regulation of the private sector and implementation of protocols across the board will help improve access to and quality of care provided by both private and public sectors.

### Vital registration system and reporting of maternal deaths for quality services

India needs to develop a system to accurately record all births and deaths. Reliable data about mortality can be generated cost-effectively and be used for improving programme inputs and to know the trends in mortality rates over time. The system of reporting maternal deaths should be strengthened, and each maternal death should be audited to improve maternal health services. Quality-assurance systems, such as the use of evidence-based protocols and criteria-based audits, need to be instituted in each facility.

### Generating the political will and advocates for maternal health

Health needs to be a priority issue for politicians, and maternal health in particular needs attention. Politicians must ensure that an adequate number of officers with technical competence are looking after maternal health consistently for longer periods. Top politicians, such as health ministers and Chief Minister at the state level and Prime Minister at the national level, should periodically review MMR and maternal health services. Women's NGOs need to take up the issue of high maternal mortality as an important issue to ensure that the Government is focused in its efforts to reduce maternal deaths. International development agencies and national-level NGOs need to join hands with professional organizations, such as FOGSI, to make maternity safer in India.

### Conclusion

India has progressed rapidly on the socioeconomic front but progress in the improvement of maternal health has been slow. Review of safe motherhood efforts in India shows that, despite major initiatives taken by the Government in the last 10 years, till recently, nearly half of all deliveries take place at home, and the coverage of antenatal care services is low. The MMR still remains at around 300-450. The challenge is how to make safe motherhood strategies in the future more successful. Strengthening EmOC should be the focus of the safe motherhood strategy, along with ensuring skilled care at all births. Policy and programmes designed to implement evidence-based strategies and detailed micro-level programme planning are needed. Monitoring effective implementation and measuring progress is essential for success. It will take at least 10-15 years of consistent, concerted and committed efforts towards improving maternal health to show results.

## References

[1] World Health Organization (2007). Maternal mortality in 2000: estimates developed by UNICEF and UNFPA. Geneva: World Health Organization.

[2] (2007). Family welfare statistics in India—2006.

[3] (1946). Report of the Health Survey and Development Committee, Government of India.

[4] (1961). Report of the Health Survey and Planning Committee, Government of India.

[5] Visaria PM (1969). The sex ratio of the population of India. Census of India 1961.

[6] Bhatia JC (1993). Levels and causes of maternal mortality in Southern India. Stud Fam Plann.

[7] Iyengar SD, Gupta V, Iyengar K. (2009). Maternal health: a case study of Rajasthan. J Health Popul Nutr.

[8] International Institute for Population Sciences (1995). National family health survey (MCH and family planning), India, 1992-93.

[9] Registrar General of India (2006). Maternal mortality in India: 1997-2003: trends, causes and risk factors; summary.

[10] Hill K, Thomas K, Abou Zahr C, Walker N, Say L, Inoue M (2007). Estimates of maternal mortality worldwide between 1990 and 2005: an assessment of available data. Lancet.

[11] India. Ministry of Health and Family Welfare National health policy 2002. http://mohfw.nic.in/np2002.htm.

[12] Alok R, Stones RW., Alok R, Stones RW (2004). Obstetric care in India policy and service delivery. Obstetric care in central India.

[13] India. Planning Commission (2002). Tenth five year plan(2002-2007). V. II. Chapter 2.

[14] Registrar General of India (2006). Maternal mortality in India: 1997-2003: trends, causes and risk factors: Table-6.

[15] National family health survey 3 (2005-2006) National fact sheet—India. http://www.nfhsindia.org/pdf/IN.pdf.

[16] National family health survey 3 (2005-2006) (2007). V. I. Chapter 8. Maternal health.

[17] National family health survey 1. Chapter 9. Maternal health. http://hetv.org/pdf/nfhs/india1/iachap9.pdf.

[18] (2007). Family welfare statistics in India—2006. Table-A.43.

[19] Health information of India—2005. Chapter 7. Manpower statistics. http://www.cbhidghs.nic.in/hia2005/chap7.asp.

[20] Bulletin of Rural Health Statistics, 2006. http://mohfw.nic.in/Bulletin%20on%20RHS%20-20March,%202007%20-%20PDF%20Version/RHS%20Bulletin%20March%202007%20-%20Tables.pdf.

[21] (2007). Family welfare statistics in India—2006. Table-A.43.1.

[22] (2007). Family welfare statistics in India—2006. Table-A.43.2.

[23] (2007). Family welfare statistics in India—2006. Table-A.43.2.

[24] Park K (2005). Health planning and management. Park's Text book of preventive and social medicine.

[25] Mavalankar D, Vora K. (2008). The changing role of auxiliary nurse midwife (ANM) in India: implications for maternal & child health (MCH).

[26] Iyer A, Jesani A, Fernandes A, Hirani S, Khanvilkar S. (1995). Women in health care: auxiliary nurse midwives.

[27] Ramani KV, Mavalankar DV. Management capacity assessment for national health programs: a study of RCH programe in Gujarat state, 2007. (Working paper no. 2007-03-02). http://www.iimahd.ernet.in/publications/data/2007-03-02_kvramani.pdf.

[28] National Child Survival and Safer Motherhood Programme (1992). Subcentre work plan. Sec. 12. Activities at the village. Sec. 13: Activities at the subcentre. Sec. 14. Module for health workers.

[29] (1997). Implementation completion report: India. Child Survival and Safe Motherhood Project.

[30] (2005). Lessons from RCH Phase I, RCH Phase II national program implementation plan.

[31] (2005). National rural health mission framework for implementation 2005-2012.

[32] National AIDS Control Organization (2007). An action plan for blood safety, 2007.

[33] Ramani KV, Mavalankar DV, Govil D. (2009). Study of blood-transfusion services in Maharashtra and Gujarat states, India. J Health Popul Nutr.

[34] India. Ministry of Health and Family Welfare (2006). *Janani Suraksha Yojana*: features & frequency asked questions and answers.

[35] Mavalankar DV, Vora KS, Ramani KV, Raman P, Sharma B, Upadhyaya M. (2009). Maternal health in Gujarat, India: a case study. J Health Popul Nutr.

[36] Padmanaban P, Raman PS, Mavalankar DV. (2009). Innovations and challenges in reducing maternal mortality in Tamil nadu, India. J Health Popul Nutr.

[37] Principal performance ratings, implementation completionreport: Washington, DC: World Bank, 2005:1. (Reportno. 30479-IN). http://www-wds.worldbank.org/external/default/WDSContentServer/WDSP/IB/2005/06/06/000012009_20050606101021/Rendered/PDF/304790rev.pdf.

[38] Shiffman J, Ved RR. (2007). The state of political priority for safe motherhood in India. Br J Obstet Gynaecol.

[39] Bhat R, Mavalankar DV, Singh PV, Singh N. (2009). Maternal healthcare financing: Gujarat's Chiranjeevi Scheme and its beneficiaries. J Health Popul Nutr.

[40] World Bank (2008). Global monitoring report 2008—MDGs and the environment: agenda for inclusive and sustainable development.

[41] Mavalankar DV, Rosenfield A (2005). Maternal mortality in resource-poor settings: policy barriers to care. Am J Public Health.

